# Fracture of Ceramic Bearing Surfaces following Total Hip Replacement: A Systematic Review

**DOI:** 10.1155/2013/157247

**Published:** 2013-06-13

**Authors:** Francesco Traina, Marcello De Fine, Alberto Di Martino, Cesare Faldini

**Affiliations:** ^1^Department of Rizzoli-Sicilia, Orthopaedic Service, The Rizzoli Institute, Bagheria, Italy; ^2^Department of Orthopaedics and Trauma Surgery, Center of Integrated Research (CIR), University Campus Bio-Medico of Rome, Rome, Italy

## Abstract

Ceramic bearing surfaces are increasingly used for total hip replacement, notwithstanding that concern is still related to ceramic brittleness and its possible mechanical failure. The aim of this systematic review is to answer three questions: (1) Are there risk factors for ceramic component fracture following total hip replacement? (2) Is it possible to perform an early diagnosis of ceramic component failure before catastrophic fracture occurs? (3) Is it possible to draw guidelines for revision surgery after ceramic components failure? A PubMed and Google Scholar search was performed and reference citations from publications identified in the literature search were reviewed. The use of 28 mm short-neck femoral head carries an increased risk of fracture. Acetabular component malposition might increase the risk of ceramic liner fractures. Synovial fluid microanalysis and CT scan are promising in early diagnosis of ceramic head and liner failure. Early revision is suggested in case of component failure; no consensus exists about the better coupling for revision surgery. Ceramic brittleness remains a major concern. Due to the increased number of ceramic on ceramic implants, more revision surgeries and reports on ceramic components failure are expected in the future. An algorithm of diagnosis and treatment for ceramic hip failure is proposed.

## 1. Introduction

Total hip replacement (THR) is one of the most successful surgical procedures and more than 285,000 interventions are carried out each year in the United States according to the Agency for Healthcare Research and Quality (http://www.ahrq.gov/). Metal on polyethylene (MOP) represent the most commonly implanted bearing surfaces up to date, whereas metal on metal (MOM) and ceramic on ceramic (COC) are less frequently used. However, MOP couplings are associated to the formation of polyethylene wear particles that can induce periprosthetic inflammatory response and osteolysis with subsequent implant failure [1–5]. Similarly, elevated serum levels of metal ions have been found in patients with MOM couplings, known to be associated to possible adverse effects such as renal toxicity or chromosomal aberrations [6–11].

In this contest, ceramic bearing surfaces are increasingly used for THR and good mid to long term outcomes have been reported, seen the outstanding tribological properties that make it a valuable alternative to MOM or MOP couplings, above all in the increasing population of younger patients undergoing THR [12–14]. In fact, the elevated scratch resistance and wettability of ceramics produce excellent fluid-film lubrication with negligible wear [15]: the biological inertness of ceramic particles avoids then the risk of periprosthetic osteolysis [1–3] and the concerns generally related to metal ions [6–11]. Due to these reasons an even greater number of COC hip prostheses could be expected in the future.

However, the main issue related to ceramic materials is the intrinsic brittleness. The hardness of ceramics hamper plastic deformation under loads, and when cyclic loads are applied over the ceramic components, microscopic imperfections such as pores or inhomogeneity of the material can act as stress risers leading to the propagation of cracks with potential component failure [16].

**Figure 1:**
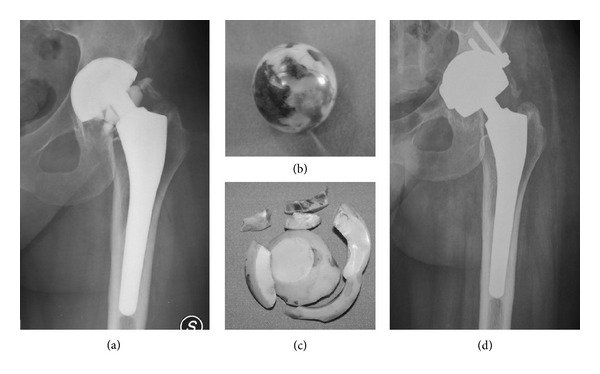
(a) Preoperative radiograph showing catastrophic failure of a ceramic liner. (b) The retrieved ceramic head grossly damaged because of contact with metal back. (c) The retrieved ceramic liner. (d) Postoperative radiograph after cup revision and bearing surfaces exchange to metal on metal coupling.

Alumina (Al_2_O_3_) and Zirconia (ZrO_2_) ceramics have been historically used for THR. With progresses in manufacturing since the late 1990s second and third generation alumina (Biolox and Biolox Forte, resp.) were available on the shelf. Newer ceramic materials have been modified to address the issue of potential component failure, and are characterized by a greater fracture toughness and lower wear rate compared to alumina and Biolox or Biolox Forte. This newer materials have been realized using additives to achieve the so called “Alumina Matrix Composite”, with the trade name of Biolox Delta [16]. However even with the implementation of newer ceramics, the risk of component failures is still present in a small percentage of patients [17, 18], and it represents a catastrophic event that inevitably requires revision surgery (Figure 1), whose results might be unpredictable in terms of implant survival and related complications [19]. 

Since little has been published concerning diagnosis and treatment of failed COC hip prostheses, the aim of this systematic review was to scrutinize the available literature in order to answer three questions: (1) Are there risk factors for ceramic component fracture following THR? (2) Is it possible to perform an early diagnosis of ceramic component failure before catastrophic fracture occurs? (3) Which is the best treatment strategy?

## 2. Material and Methods

The scientific databases were accessed on to identify papers dealing with diagnosis and treatment of ceramic component fractures. Considering the small number of publications on this subject, exclusion criteria about the manufacture and type of ceramic components used were not supplied. For the same reason case reports were also taken into account. Therefore, the only inclusion criterion was the report of a ceramic component fracture following THR.

**Figure 2:**
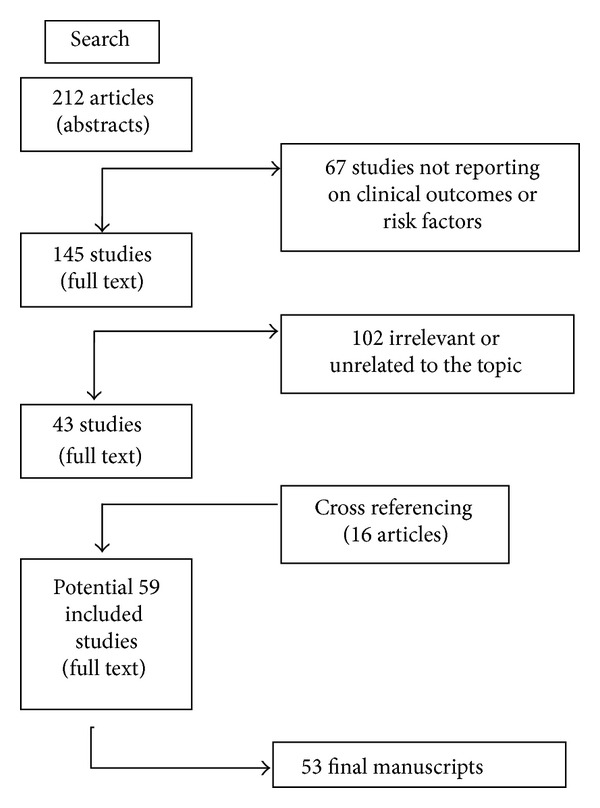
Diagram showing the process of manuscripts selection.

We performed a search using the keywords “ceramic”, “alumina”, and “total hip replacement/hip prosthesis” in combination with “failure”, “fracture”, “debris”, “diagnosis”, “revision surgery”, “component breakage”, “head”, and “risk factors” with no limit regarding the year of publication. The following databases were accessed on June 1, 2012: PubMed (http://www.ncbi.nlm.nih.gov/sites/entrez/); Ovid (http://www.ovid.com/); Cochrane Reviews (http://www.cochrane.org/reviews/); and Google Scholar. Given the linguistic capabilities of the research team, we considered publications in English and Italian (Figure 2). All journals were considered. Literature references of the selected papers were also checked in order to find further relevant publications. Two authors (M. D. Fine and A. D. Martino) read the abstract and excluded the articles that were considered unrelated to the topic of the study. When abstract was not available (such as in case reports) the title of the paper was used to judge its relevance. In case of doubt about inclusion of an article, the senior author (C. Faldini) made the decision.

Three of us (A. Di Martino, M. De Fine, and F. Traina) extracted from the retained articles information regarding the following fields: (1) risk factors for ceramic component fractures; (2) early diagnosis of ceramic component fractures; and (3) treatment strategies and therapeutic algorithms for revision surgery; these searches yielded 212 articles. Two authors (M. De Fine and A. Di Martino) read the abstract or the title of each paper. From the total of 212 articles, we excluded 67 not reporting on risk factors or indications for surgery in the abstract such as letters to the editor, technical descriptions, or because the article was not published in peer-reviewed journals, leaving 145 articles.

Based on the abstract or the title we excluded 102 articles we judged irrelevant because they were unrelated to the topic of the study. In addition, the search was extended by screening the reference list of all the articles. This cross-referencing process added further 16 articles to the 43 previously identified. For the remaining 59 articles we obtained full-text versions. To avoid bias in including the articles, the publications selected were examined and discussed by all the co-authors. After this further selection, 53 publications relevant to the topic at hand were included (Figure 2). There were one randomized multicenter trial, two case-control studies, 23 retrospective case-series, 25 case reports, one review, and one laboratory study.

## 3. Results and Discussion 

Ceramic brittleness remain an unresolved question and nowadays surgeons need guidelines about how to diagnose and treat fractures of ceramic bearing surfaces following THR.

This systematic review was performed aiming to answer three main questions: (1) Are there risk factors for ceramic component fracture following THR? (2) Is it possible to perform an early diagnosis of ceramic component failure before catastrophic fracture occurs? (3) Which is the best treatment strategy?

### 3.1. Risk Factors

Risk factors were analyzed separately for head and liner fractures.

Ceramic head fracture is a catastrophic event and several cases of fractured heads are reported in scientific databases [20–30, 35–52]. The majority of these manuscripts are case reports and retrieved analysis of fractured heads [28, 29, 35–41, 43–45, 48–51]. The remaining studies reported on the incidence of fractured ceramic heads in retrospective case-series on the mid to long term outcomes of COC hip prostheses [20–22, 24–28, 30, 42, 46]. A trauma was involved in the generation of fractures in 7 reports [24, 26, 27, 35, 42, 46, 48]. Only two papers specifically focused on risk factors for ceramic head fractures. Koo et al. found 5 head fractures among 367 COC hip prostheses using third generation 28 mm heads [23]. All fractured components were short-neck heads and in all cases the fracture involved the circumferential portion of the head near to the edge of the head bore. The authors postulated that using 28 mm heads the distance between the corner of the head bore and the outer surface of the ceramic head is smaller in comparison with the medium and long neck designs, thereby facilitating the propagation of cracks. These findings are in agreement with the work of Callaway et al. [29], in which a greater risk of fracture was also identified for second generation 28 mm short-neck heads. On the contrary, in two manuscripts it was hypothesized that long neck designs could facilitate head fractures because the increased distance between the edge of the head bore and the outer surface of the head itself increases the tensile stresses at the taper-bore junction [22, 45]. However, reported data were not sufficient to support this theory. Based on the available literature, the only factor associated to the risk of ceramic head fracture is the use of short neck 28 mm heads. Reported rates of ceramic head fractures and generation of material used, are reported in Table 1.

Ceramic liner fracture is generally a subtle and underestimated event, and is not directly related to traumas. The occurrence of ceramic liner fracture is reported to be between 0,013% [53] and 1,1% of patients undergoing COC THR [18]. Even in this case, the introduction of newer ceramic materials did not eliminate the risk of a catastrophic failure [18]. We found 21 published manuscripts reporting on fractures of ceramic liners [17, 18, 31–34, 46, 54–67]. There were 8 case reports [17, 54, 57–61, 65], 8 retrospective case-series dealing with the outcomes of COC hip prostheses [31, 32, 46, 55, 56, 62–64, 66], one case-control study [34], one laboratory study [67], and one multicenter trial [18]. Most of these reports regarded the use of sandwich-type liners [17, 55, 56, 60, 62–66], in which a polyethylene layer is interposed between a thinner than usual ceramic liner and the metal back, in the effort to reduce the stiffness mismatch between ceramic and metal back. The literature clearly advises against the use of such hybrid devices; that should be considered different with respect to traditional ceramic liners in terms of design, therefore we did not take into account the conclusions of these manuscripts. In all but one of the remaining manuscripts (Table 2), the authors anecdotally reported sporadic cases of liner fractures, but a clear analysis of risk factors eventually related to failure was not performed [18, 31–33, 46, 54, 57–59, 61]. The most accepted hypothesis as a causative factor for implant failure is the cyclic impingement between the neck of the stem and the acetabular component. This mechanism could determine a head subluxation with peak stresses on the opposite side of the liner, thus determining its fracture. In this contest, the relative position of the acetabular component in respect to the stem and pelvis itself could play an important role in determining this conflict; however, the number of reported cases is too small to draw definitive conclusions. The only case-control study on the subject compared 26 failed COC hip prostheses revised because of ceramic liner fracture with 49 age-matched well-functioning COC hip prostheses [34]. The populations were comparable in terms of demographics, type of ceramic components used and implant position; CT scan of the pelvis was available in 22 out of 26 cases of fractured ceramic liner and in all cases in the control group. A greater number of cups placed outside the optimal range of cup anteversion was found in the failure group (*P* = 0,03) with an audible noise detectable in 21 patients (80,7%) in the fracture group, compared to only 3 cases (6,1%) observed in the non-fracture group (*P* = 0,001). Data extracted from this series support the hypothesis that neck-to-cup impingement with head subluxation and edge load on the opposite side of the liner could produce a liner fracture. Since the evaluation of cup positioning in patients with suspect liner failure is suggested, a CT scan of the pelvis should be performed to evaluate cup position on the axial plane, and contemporarily to ascertain the presence of ceramic fragments eventually not visible with traditional X-ray. 

It has also been supposed that chipping because of misalignment of the liner during insertion into the cup could be the cause of failure in many instances [18]. McAuley et al. tested this hypothesis by using a laboratory model and demonstrated that misalignment of the liner during impaction into the acetabular component does significantly increase the risk of liner fractures [67]. On the basis of the aforementioned papers cup malposition on the axial plane and misalignment of the liner during insertion was found to be the only two relevant factors affecting the risk of liner fractures.

### 3.2. Diagnosis

No papers dealing with early diagnosis of ceramic head fractures were found. On the contrary, two studies suggested that synovial fluid microanalysis after hip needle aspiration as a valuable tool for early diagnosis of ceramic liner fractures [33, 68]. Toni et al. evaluating eight liner fractures among 3710 COC hip prostheses found a correlation between noise following total hip replacement and ceramic liner fracture. The same authors further demonstrated that the presence of ceramic fragments greater than 5 *μ*m after synovial fluid examination is strongly associated with the presence of liner fracture [33]. In the second study the synovial fluid harvested from 12 well-functioning COC hip prostheses was used to set the physiological amount of ceramic particles in normal COC implants and then the results of hip needle aspiration performed in 39 COC hips followed for noise or discomfort was compared with that of seven COC hips scheduled for revision surgery for reason unrelated to the ceramic coupling [68]. Hip aspiration was performed under sterile conditions using C-Arm intensifier or ultrasound. Synovial fluid was dropped onto a polycarbonate filter and particles were isolated adding sodium hypochlorite. Finally scanning electron microscopy allows particles measurement [68, 69].

The authors defined the presence of at least 11 ceramic particles smaller than 3 *μ*m or at least one ceramic particle larger than 3 *μ*m per each 90 *μ*m^2^ field of observation as a strong level of ceramic liner damage, and demonstrated that synovial fluid microanalysis had 100% sensitivity and 88% specificity in predicting ceramic liner fractures in case of strong presence of ceramic particles. Despite further study should confirm this observation on larger populations, synovial fluid microanalysis seems to be a valuable tool for early diagnosis of ceramic liner fracture. 

The noise could be considered as predictive of ceramic liner failure in THR [33, 34] and, although further studies would be needed to definitively confirm this hypothesis, the presence of a noisy hip following THR should raise the suspicion of liner fracture and require appropriate investigations such as CT scan [34].

### 3.3. Treatment Strategy

Revision surgery for fractured ceramic components can be troublesome, and could be associated to poor results [70]. In fact, it has been speculated that the presence of sharp ceramic fragments retained in the artificial joint space could act as an abrasive paste affecting the performance of the new articular coupling. Besides, concerns exist about the reimplantation of a new head on a previously used morse taper because of the presumed higher risk of new fracture due to fretting corrosion of the morse taper [19].

At present, there is no consensus about the best strategy to address revision surgery in patients with failure of ceramic implant. Revision surgery for fractured ceramic components should be carried out urgently in order to reduce the risk that ceramic particles further damage the metal taper [19]. Rest and avoidance of weight bearing until surgery are advisable with the aim to reduce the diffusion of ceramic particles and damages to the neck of the stem and to the metal cup. Surgery should always include an extensive synoviectomy and thorough irrigation of the articular space, since the complete elimination of ceramic fragments is of paramount importance to increase the survivorship of the new articulation [19, 70, 71]. In a retrospective study on the outcomes of 105 revisions performed for fractured ceramic heads Allain et al. found a 31% rate of failure at a mean 3.5 years follow-up [70]. The authors conclude that survival rate was significantly decreased when complete synoviectomy was not performed.

Another controversial technical issue in revision surgery is the choice of the best articular coupling to use. Sharma et al. [71] followed-up eight hips that were revised to metal on polyethylene articulations using cobalt chromium heads after fracture of ceramic heads. The authors found no revision due to osteolysis or aseptic loosening at a mean 10.5 years follow-up. Other authors advise the use of ceramic on ceramic or ceramic on polyethylene (COP) couplings, since the elevated scratch resistance of the ceramic could reduce the risk of third body wear [19]. Although no sufficient data are available to clearly identify the best coupling, COC or COP seems to reduce the risk of third body wear.

Since the report of Pulliam and Trousdale [72] the reimplantation of a new ceramic head on a previously used morse taper is considered hazardous because the risk of fretting corrosion could rapidly lead to a new ceramic head fracture. However, Hannouche et al. [46] evaluated the results of revision surgery for fractured ceramic heads and found no fractures among 61 ceramic heads that were re-implanted on a non-revised titanium morse taper at a mean of 88 ± 65 months. The authors suggested that the original morse taper can be used safely if no major damages of the taper are evident during surgery. On the basis of the available literature it is not possible to draw definitive conclusions about this question, because although damaged morse tapers could significantly increase the risk of re-fracture using ceramic heads, the routinary explantation of well-fixed stems could be very troublesome. 

THR with COC couplings are expected to increase progressively in the next future; however, despite the improvement in materials manufacture, ceramic brittleness is still a major concern and surgeons should be aware of current standards of evaluation and management of the failed COC hip arthroplasty. This systematic review was performed to answer three questions considered relevant in addressing the issue of the failed COC THR, and, namely, determination of risk factors for ceramic component fracture, definition of standards for early diagnosis of ceramic component failure and for updated management strategies.

**Figure 3:**
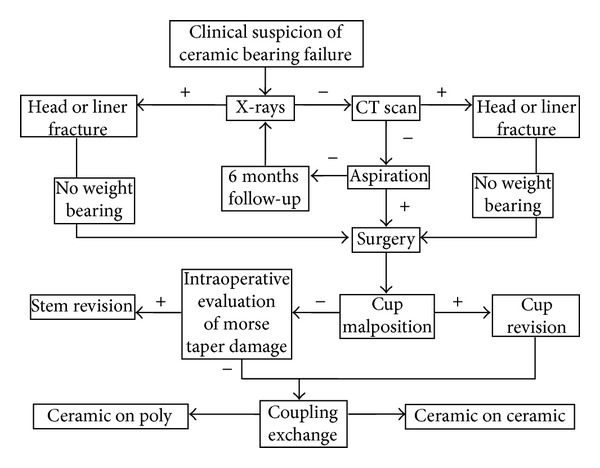
Algorithm with guidelines for the diagnosis and treatment of fractured ceramic bearings following THR.

When addressing the suspicion of a rupture of ceramic components of hip arthroplasty (Figure 3), the first diagnostic step is as usual the performance of standard X-rays; in most cases an adjunctive CT scan is required to refine the diagnosis and better characterize the mutual relationship between the cup and the stem. Current knowledge consider the use of short neck 28 mm heads as the only factor that significantly affects the risk of ceramic head fracture, and malposition of the cup on the axial plane and misalignment of the liner during insertion as the only two relevant factors affecting the risk of liner fractures. Synovial fluid microanalysis seems to be a valuable tool for early diagnosis of ceramic liner fracture and the presence of a noisy hip following THR should raise the suspicion of liner fractures; however, data on synovial fluid aspiration reliability should be confirmed on larger cohorts of patients. Once the diagnosis of ceramic component fracture is done, revision surgery is required. It is not possible to draw definitive conclusions about the best treatment strategy, however during revision surgery an extensive synoviectomy and thorough irrigation of the articular space are mandatory, while COC or COP couplings are both considered as viable options to reduce the risk of third body wear of revised implants.

## Figures and Tables

**Table 1:** Rate of ceramic head fractures in the literature (the percentage was calculated on the basis of nontraumatic head fractures).

Author	Type of ceramic	No. of hips	No. of fractures (traumatic)	%
Lee et al. [20]	3rd generation (BIOLOX FORTE)	86	2 (1)	1.1
Mannan et al. [21]	Unspecified (surgery 1989–1992)	100	2	2
Aldrian et al. [22]	2nd generation (BIOLOX)	107	3	2.8
Koo et al. [23]	3rd generation (BIOLOX FORTE)	367	5	1.4
Fayard et al. [24]	Unspecified (surgery 1991-1992)	102	2 (2)	0
Park et al. [25]	3rd generation (BIOLOX FORTE)	357	2	0.6
Yoo et al. [26]	3rd generation (BIOLOX FORTE)	72	2 (1)	1.4
Jeong et al. [27]	3rd generation (BIOLOX FORTE)	100	1 (1)	0
Toni et al. [28]	1st generation (ALUMINA)	82	2	2.4
Callaway et al. [29]	Unspecified	184	4	2.2
Nizard et al. [30]	Unspecified (surgery 1977–1979)	87	5	5.7

**Table 2:** Rate of ceramic liner fractures in the literature (nontraumatic in all cases).

Author	Type of ceramic	No. of hips	No. of fractures	%
Hamilton et al. [18]	4th generation (BIOLOX DELTA)	157	2	1.3
Traina et al. [31]	3rd generation (BIOLOX FORTE)	61	1	1.6
Choi et al. [32]	3rd generation (BIOLOX FORTE)	173	1	0.6
Toni et al. [33]	Unspecified (surgery 1993–2004)	3710	8	0.2
Traina et al. [34]	Unspecified (surgery 2000–2010)	6648	22	0.3
